# Co-Pyrolysis of Sewage Sludge and Wetland Biomass Waste for Biochar Production: Behaviors of Phosphorus and Heavy Metals

**DOI:** 10.3390/ijerph19052818

**Published:** 2022-02-28

**Authors:** Ilham Gbouri, Fan Yu, Xutong Wang, Junxia Wang, Xiaoqiang Cui, Yanjun Hu, Beibei Yan, Guanyi Chen

**Affiliations:** 1 Tianjin Key Laboratory of Biomass Waste Utilization, School of Environmental Science and Engineering, Tianjin University, Tianjin 300072, China; 6219000180@tju.edu.cn (I.G.); 1112102034@zjut.edu.cn (F.Y.); wangxutong@tju.edu.cn (X.W.); wangjunxia@tju.edu.cn (J.W.); yanbeibei@tju.edu.cn (B.Y.); chen@tju.edu.cn (G.C.); 2 Institute of Energy and Power Engineering, Zhejiang University of Technology, Hangzhou 310023, China; huyanjun@zjut.edu.cn; 3 School of Mechanical Engineering, Tianjin University of Commerce, Tianjin 300134, China

**Keywords:** sewage sludge, wetland plant, co-pyrolysis, phosphorus, heavy metals

## Abstract

Large amounts of sewage sludge (SS) and wetland plant wastes are generated in the wastewater treatment system worldwide. The conversion of these solid wastes into biochar through co-pyrolysis could be a promising resource utilization scheme. In this study, biochar was prepared by co-pyrolysis of SS and reed (*Phragmites australis*, RD) using a modified muffle furnace device under different temperatures (300, 500, and 700 °C) and with different mixing ratios (25, 50, and 75 wt.% RD). The physicochemical properties of biochar and the transformation behaviors of phosphorus (P) and heavy metals during the co-pyrolysis process were studied. Compared with single SS pyrolysis, the biochar derived from SS-RD co-pyrolysis had lower yield and ash content, higher pH, C content, and aromatic structure. The addition of RD could reduce the total P content of biochar and promote the transformation from non-apatite inorganic phosphorus (NAIP) to apatite phosphorus (AP). In addition, co-pyrolysis also reduced the content and toxicity of heavy metals in biochar. Therefore, co-pyrolysis could be a promising strategy to achieve the simultaneous treatment of SS and RD, as well as the production of value-added biochar.

## 1. Introduction

Sewage sludge (SS) is a byproduct of wastewater treatment plants (WWTPs) consisting of a considerable amount of inorganic nutrients (i.e., phosphorus, nitrogen, and potassium) [1]. The annual generation amount of sewage sludge in China has reached approximately 39.04 million tons in 2019 with a moisture content of up to 80%, and more increases are expected in the next few years based on economic and population growth [2,3]. Sewage sludge has good potential to be applied to agricultural lands as it can improve soil fertility and promote organic carbon storage [4]. Nevertheless, sewage sludge generated from WWTPs contained some toxic substances such as heavy metals, polycyclic aromatic hydrocarbons, and pathogenic microorganisms, which could increase the risk of secondary pollution [5,6]. Therefore, it is necessary to provide a suitable approach for sewage sludge disposal.

The common disposal methods of sewage sludge include landfill, incineration, and land application [7]. Landfill and land application have been restricted due to the poisonous leachate and limited soil area, while incineration is limited by high operational cost and emission of hazardous gases [8]. Pyrolysis (the process of thermochemical decomposition of organic matter under anoxic conditions) is a promising alternative as it can not only kill the pathogens and parasites contained in sewage sludge, but also produce value-added bioenergy (bio-oil and bio-gas) [7,9]. The remaining solid residue, biochar, has good potential to improve soil quality via increasing contents of soil nutrients (i.e., N, P, and K), soil microbial biomass, and soil pH [10]. The integration of sewage sludge biochar into agricultural soil can enhance soil fertility by promoting nutrient supplementation, improving soil aeration and cation exchange capacity [11]. Besides pyrolysis, co-pyrolysis of sewage sludge and biomass wastes such as crop straw and wood chips was also employed as a promising method to produce biochar with better characteristics (e.g., high stability, moderate pH, and high available phosphorus content) and low metal toxicity [12]. 

Co-pyrolysis of sewage sludge and biomass waste such as cereal straw, wood chips, sawdust, and grain husks was investigated by the researchers [7,13,14]. Co-pyrolysis with bamboo sawdust produced biochars with higher stability [7], while blending rice husk with the sewage sludge for co-pyrolysis improved metal stability in the biochars, especially at 700 °C [7,13]. Yin et al. [13] reported that the co-pyrolysis of sewage sludge with organic additives (i.e., reed straw, brewers’ spent grain, and sawdust) increased the carbon (C) content of biochar while decreasing the pH, ash content, electrical conductivity, H/C, and O/C of biochar, which were beneficial to soil improvement and C sequestration. In addition, the behaviors of P and heavy metals during the co-pyrolysis of sewage sludge and biomass have drawn increasing attention. Zhao et al. [15] found that K, Cl, Ca, or Mg elements in cotton stem could combine with phosphorus during co-pyrolysis, thus promoting the transformation of non-apatite inorganic phosphorus (NAIP) to apatite phosphorus (AP). Jin et al. [16] pointed out that the addition of bamboo sawdust to sewage sludge resulted in the transformation of heavy metals from active states (F1 + F2) to a potentially active state (F3) and a stable state (F4), which reduced the toxicity of heavy metals in the derived biochars. However, all of the biomass wastes were not derived from the wastewater treatment system. Consequently, it would generate more transportation costs for the collection of sewage sludge and biomass waste during the biochar production process.

Constructed wetlands (CWs) are designed to remove pollutants from contaminated water through physical, chemical, and biological treatment processes [17]. Considering the discharge standards of pollutants for municipal wastewater treatment plants, the treated water from WWTPs might not conform with the environmental quality standards for surface water, and thus constructed wetland was extensively used as a general treatment technique for the purification of effluent from WWTPs. However, the wetland plants that are continuously produced during the water purification process needed to be appropriately disposed of to ensure the targeted treatment efficiency [18]. Hence, co-pyrolysis could be a potential strategy for the simultaneous disposal of wetland plants and sewage sludge in the integrated wastewater treatment system. However, no study investigated the feasibility of combining wetland plants and sewage sludge for biochar preparation, and the fates of phosphorous and heavy metals during the co-pyrolysis process remain largely unknown.

In this study, sewage sludge and a typical wetland plant (*Phragmites australis*) were co-pyrolyzed with different mixing ratios at different temperatures. The objectives were to: (1) investigate the characteristics of composite biochar; (2) determine the transformation of phosphorus during the co-pyrolysis process; and (3) study the speciation of the heavy metal in the derived biochars.

## 2. Materials and Methods

### 2.1. Material Preparation

Sewage sludge was obtained from a wastewater treatment plant with a daily processing capacity of 30,000 m^3^ located in Tianjin, China, where an anaerobic-anoxic-oxic (A^2^/O) system was operated for the residential wastewater treatment. The SS used in this experiment was treated by mechanical dehydration in the wastewater treatment plant, and its initial moisture content was about 80%. The reed (*Phragmites australis*, RD) was collected from a constructed wetland (horizontal subsurface flow-surface flow) used for treating WWTP effluent in Tianjin, China. The SS and RD were sun-dried for one week and dried in the oven at 80 °C for 24 h. The dried samples were ground into powder (≤0.45 mm). Sieved SS and RD were mixed together, where the percentages of RD were 25, 50, and 75 wt.%, respectively.

### 2.2. Thermogravimetric Analysis

The thermogravimetric (TG) behavior of SS, RD, and their relative mixture was investigated using a thermogravimetric analyzer (Diamond TG/DTA) from PerkinElmer. Each sample (10 ± 0.5 mg) was heated from room temperature to 800 °C using heating rates of 10 °C min^−1^. The carrier gas used in the TG analysis was N_2_ and its flow rate remained constant at 50 mL min^−1^. Derivative thermogravimetric (DTG) represented the change rate of weight during pyrolysis. To investigate the interactions between SS and RD during the co-pyrolysis, the theoretical TG/DTG curves of SS-RD blends were calculated. The calculated curves were obtained from the equation below, representing the sum of separate feedstock behavior in the mixture.

(1) $$W_{b}=m*W_{S}+\left(1-m\right)*W_{R}$$

where $W_{S}$ and $W_{R}$ refer to the mass loss of individual SS and RD under the same operational condition, respectively, $W_{b}$ represents the theoretical mass loss of the SS-RD blend, and m is the mass fraction of SS in the mixture.

### 2.3. Preparation of Biochar

The sewage sludge, *Phragmites australis*, and their relative mixture were placed in a modified muffle furnace using ceramic crucibles. The furnace was purged with N_2_ prior to use, and then N_2_ flow was continuously supplied for each run to ensure the oxygen-free conditions. The furnace was heated to three different temperatures (300, 500, and 700 °C) at a rate of 10 °C min^−1^ under oxygen-free conditions. The furnace naturally cooled to room temperature after maintaining the target temperature for 2 h. The biochar production was performed in triplicate at each temperature, and all the triplicated samples were mixed well to obtain the final sample. The biochar derived from SS, RD, and SS-RD blends were named SBs, RBs, and SRBs, respectively. All of the biochar samples were ground to pass through a 0.25-mm sieve prior to use. 

To study the interactions between SS and RD during the co-pyrolysis, the theoretical biochar yields from pyrolysis of the SS-RD blend were calculated as follows:

(2) $$Y_{t}=m*Y_{S}+\left(1-m\right)*Y_{R}$$

where $Y_{S}$ and $Y_{R}$ refer to the biochar yield from individual pyrolysis of SS and RD under the same operational condition, respectively, $Y_{t}$ represents the theoretical biochar yield from pyrolysis of SS-RD blend, and m is the mass fraction of SS in the mixture. $Deviation\left(∆\right)$ between the theoretical and experimental biochar yield was calculated by the equation below:

(3) $$Deviation\left(∆\right)=\left(Y_{e}-Y_{t}\right)/Y_{t}*100\%$$

where $Y_{e}$ and $Y_{t}$ represent the experimental and theoretical biochar yield from pyrolysis of SS-RD blend, respectively.

### 2.4. Characterization of Biochar

The elemental compositions (C, H, and N) of raw material and their derived biochars were analyzed using an elemental analyzer (Flash-EA112, Thermo Finnigan, California, USA), and oxygen contents were calculated by the difference method. The samples were mixed with KBr and pressed into thin sheet by a mechanical device for Fourier transform infrared spectra (FTIR) analysis by an FTIR spectrophotometer (Bruker, Tensor 27, Karlsruhe, Germany) in the range of 400–4000 cm^−1^. Mineral composition in the sample was measured by X-ray diffraction (XRD, PANalytical Empyrean, Almelo, Netherlands) at 40 kV and 40 mA over the range from 5° to 75° with Cu-Kα radiation. In order to analyze the ash content, the samples were placed in ceramic pots and heated in a muffle furnace under 750 °C for 5 h.

### 2.5. Phosphorous Analysis

The Standards Measurements and Testing protocol of the European commission (SMT) [15] was applied to determine the different phosphorus (P) species in raw material and the transformation of phosphorus fractions under different pyrolysis temperatures. All the extractions were performed in triplicate including total phosphorus (TP), inorganic phosphorus (IP), organic phosphorus (OP), apatite phosphorus (AP), and non-apatite inorganic phosphorus (NAIP). The contents of P in different forms were measured using the molybdenum blue colorimetric method with a UV-visible spectrophotometer (UV-6000PC, METASH).

### 2.6. Determination of Heavy Metals

The distribution of heavy metals in the raw material and biochar samples were sequentially extracted using the modified three-step sequential extraction procedure provided by the European Communities Bureau of Reference (BCR) [19]. Briefly, 0.50 g of the dried sample and a certain amount of the extractant (20 mL of 0.11 M acetic acid, 20 mL of 0.5 M hydroxylamine hydrochloride, 5 mL of 30% hydrogen peroxide, and 25 mL of 1.0 M ammonia acetate) were added to a 50 mL polypropylene centrifuge tube. The tubes were shaken horizontally at 25 °C for 16 h and then centrifuged at 4000 rpm for 20 min. The supernatant obtained was filtered through a 0.45 µm filter, stored at 4 °C and detected. After each extraction, the remaining residue was mixed with 20 mL of deionized water, placed for centrifugation at 4000 rpm for 20 min, decanted and subjected to the next extraction experiment. The forms of heavy metals in the filtrate extracted by acetic acid, hydroxylamine hydrochloride, and hydrogen peroxide-ammonia acetate were acid-soluble (exchangeable) fraction (F1), reducible state (F2), and oxidizable state (F3), respectively. The final residue state (F4) extraction step was the same as the detection step of the heavy metal concentration in the sample [20]. The concentration of heavy metals (i.e., Cr, Ni, Cu, Zn, and Pb) was measured using an inductively coupled plasma optical emission spectrometer (ICP-OES; Prodigy 7, Teledyne Leeman Labs, Hudson, NH). The standardization was performed with certified reference material of these heavy metals (SPEX CertiPrep, USA), and the blanks were included in each batch of samples.

### 2.7. Statistical Analysis 

A statistical analysis was performed by the SPSS 22.0, and the standard deviation was obtained by descriptive statistics. An analysis of variance (ANOVA) was performed to analyze how two factors (temperature and blending ratio) affected the transformation of P in the biochar, and a post-hoc analysis was performed using Tukey’s test. Differences were considered to be statistically significant when *p* < 0.05. 

## 3. Results and Discussion

### 3.1. Thermogravimetric Analysis of Sewage Sludge (SS) and Reed (RD)

**Figure 1:**
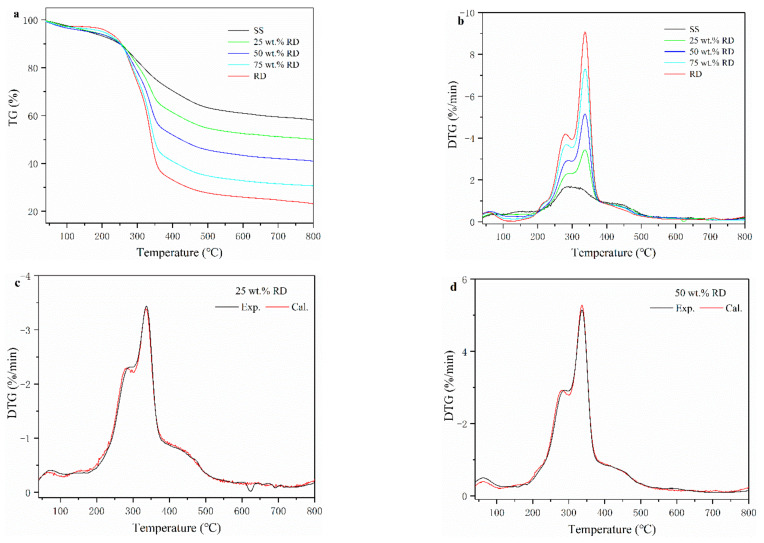

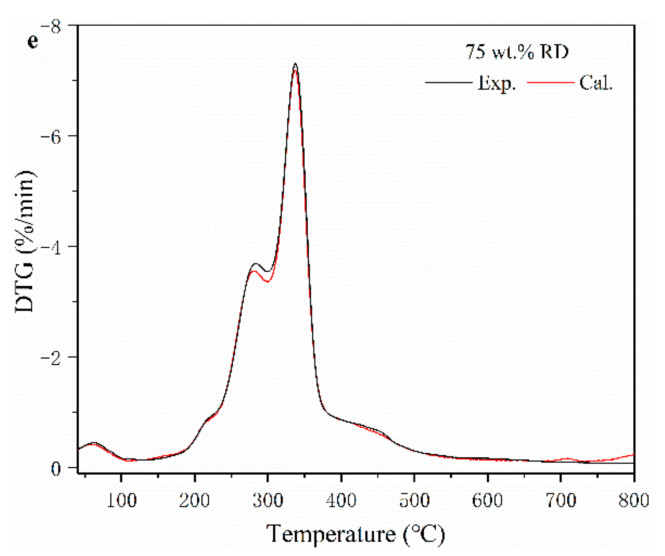
Thermogravimetric (TG) (**a**) and derivative thermogravimetric (DTG) (**b**) curves of SS, RD, and their mixture. (**c**–**e**) experimental and calculated DTG curves of SS-RD blends.

Thermogravimetric (TG) and derivative thermogravimetric (DTG) curves of SS, RD, and SS-RD mixtures are shown in Figure 1a,b. According to the DTG curve, the pyrolysis process of SS can be divided into three stages. At the first stage (below 150 °C), the weight loss of SS was 4.46% due to the release of internal water and the decomposition of a small number of weakly bonded functional groups [21]. The devolatilization of SS mainly occurred at the second stage (150–550 °C), with a weight loss rate of 33.7% from the decomposition of organic compounds (e.g., cellulose, polysaccharides, carboxylate lipids, and proteins) in the sludge into small molecular gases (such as CO_2_, CH_4,_ and H_2_) and coagulable volatiles (tar) [22]. The weight loss in the third stage (above 550 °C) of sludge pyrolysis was 3.91%, due to the aromatization of organic matter structure in char and the decomposition of inorganic salts (mainly carbonate) [23]. The pyrolysis process of RD was quite different from that of SS. The weight loss of RD (2.86%) was lower than that of SS (4.46%) in the first stage, indicating that the content of bound water in RD was lower than that of SS. In the devolatilization stage, SS had a wide peak with a maximum weight loss rate of 1.72 % min^−^^1^ (282 °C), while RD had two parallel peaks with a weight loss rate of 4.24 % min^−1^ (275 °C) and 9.24 % min^−1^ (345 °C), respectively. This was because the main components of RD were hemicellulose and cellulose, and their decomposition temperatures ranged from 220–315 °C and 315–400 °C, respectively [24]. At the termination pyrolysis temperature, the total mass loss of SS and RD accounted for 42.0% and 77.1%, respectively. The higher mass loss of RD was mainly caused by the higher volatiles and lower ash content (Table S1).

The TG-DTG curves of SS-RD blends were located between the TG-DTG curves of two single samples (Figure 1a,b), which was consistent with the results of other biomass and sludge mixings, such as rice husk and cotton stalks [25,26]. The final weight loss of 25, 50, and 75 wt.% RD was 50.2%, 59.1% and 69.4%, respectively. In order to evaluate the relationship between SS and RD during pyrolysis, the differences of experimental and calculated DGT curves of SS-RD blends were compared (Figure 1c–e). In the devolatilization stage, the decomposition peaks of hemicellulose and cellulose (275 °C and 345 °C) also existed in the SS-RD blends, which was similar to the DGT curve of RD. There was a certain deviation in the peak value and the corresponding temperature between the calculated and experimental DTG curves. In addition, the peak shoulder value of the calculated DTG curve (~300 °C) was lower than that of the experimental value, indicating that the interaction between SS and RD mainly occurred during the decomposition of hemicellulose. Correspondingly, this synergistic effect may also affect biochar production as well as the behavior of phosphorus and heavy metals.

### 3.2. Biochars

#### 3.2.1. Biochar Yield

**Figure 2:**
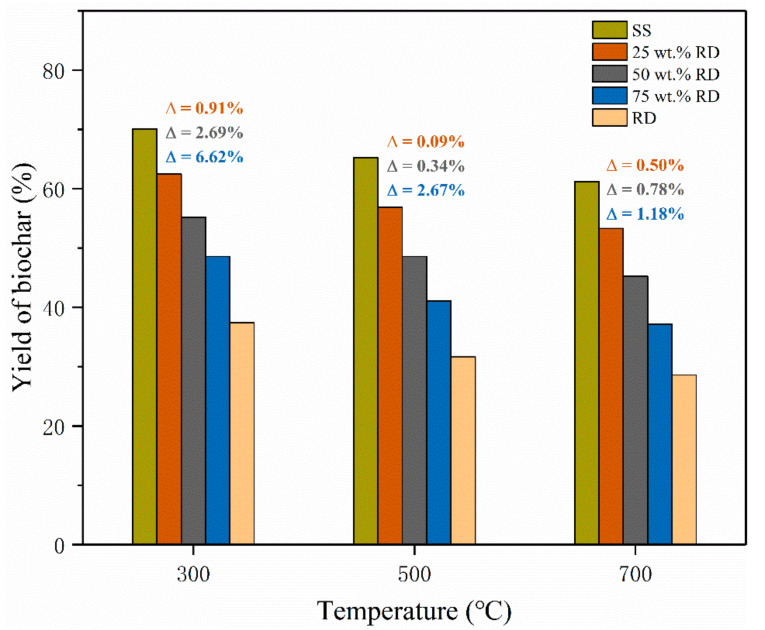
Yield of biochar from the pyrolysis of SS, RD, and SS-RD blends. (Note: ∆ was the deviation between the experimental value and the theoretical value, and the calculation method was shown in Equation (3)).

The biochar yield from SS pyrolysis decreased from 70.1% to 61.2% with increasing temperature from 300 to 700 °C, and a similar trend was observed on the biochar derived from SS-RD blends (Figure 2). The yield of solid products from RD pyrolysis (28.6–37.4%) was much lower than that from SS-derived biochar (61.2–70.1%) at the same temperature, so the yield of biochar derived from the SS-RD blend was lower than that derived from SS. In order to illustrate the interaction between SS and RD, the theoretical SS-RD biochar yield was calculated and compared with experimental values. The experimental yields of SS-RD biochar elevated by 0.09–6.62% as compared with the calculated values (Figure 2), indicating the synergistic effect of SS and RD on the production of biochar. This synergistic effect was also found for the co-pyrolysis of wood and plastics in previous studies [27,28]. The deviation between the experimental and calculated biochar yields reduced at high pyrolysis temperatures (500 and 700 °C), elucidating that the synergistic effect on biochar production was suppressed by high pyrolysis temperature. It is noteworthy that this deviation enlarged with elevating the RD addition amount, which indicated that increasing RD content could promote the synergistic effect on biochar production. Therefore, a high RD addition amount and low co-pyrolysis temperature are beneficial to the promotion of a synergistic effect on biochar production. 

#### 3.2.2. Biochar Characteristics

The basic physicochemical properties of biochars derived from the pyrolysis of SS, RD, and SS-RD blends are shown in Table S1. The pH values of SS and RD were 6.90 and 5.10, respectively. With the increase of pyrolysis temperature, the pH values of SBs and RBs increased from 7.06 and 7.16 to 9.69 and 9.14, respectively, mainly due to the decomposition of the acid functional groups and the accumulation of alkali metal salts [29]. The pH value of SRBs was higher than that of SBs and RBs at the same temperature, because the addition of RD promoted the conversion of metal compounds to alkali metal salts and the decomposition of functional groups [5]. With the increase of RD addition from 25 to 75 wt.%, the pH value of SRBs increased from 7.22–10.2 to 8.96–11.7, indicating that the promotion effect was enhanced with the increase of the RD addition. The ash content of SBs increased from 72.6% to 81.4% with the increase of pyrolysis temperature. The content of C in SBs decreased from 17.7% to 14.2%, while that in RBs increased from 63.2% to 73.5%, which was consistent with the results of previous studies [30,31]. Because the mineral content in SS was much higher than that in RD, the mineral enrichment degree of SBs was greater than the carbonization degree [31]. Compared with SBs, ash content decreased and C content increased in SRBs (Table S1), suggesting that the co-pyrolysis of SS and RD contributed to the reduction of inorganic salts and the enhancement of carbon fixation in biochar. At the same temperature, the C content in SRBs increased with the increase of RD addition since the content of C in RD (45.1%) was much higher than that in SS (21.2%). The values of H/C and (O+N)/C gradually decreased with the increase of temperature, indicating that the higher pyrolysis temperature leads to more aromatic structures and less polar surfaces of biochar [32]. The H/C value of SRBs (0.20–0.80) was lower than that of SBs (0.37–2.30) derived at the same temperature (Table S1), indicating that the co-pyrolysis of SS and RD could increase the aromatic structure of biochar, which was consistent with the results of the co-pyrolysis of sludge and rice husk [33].

**Figure 3:**
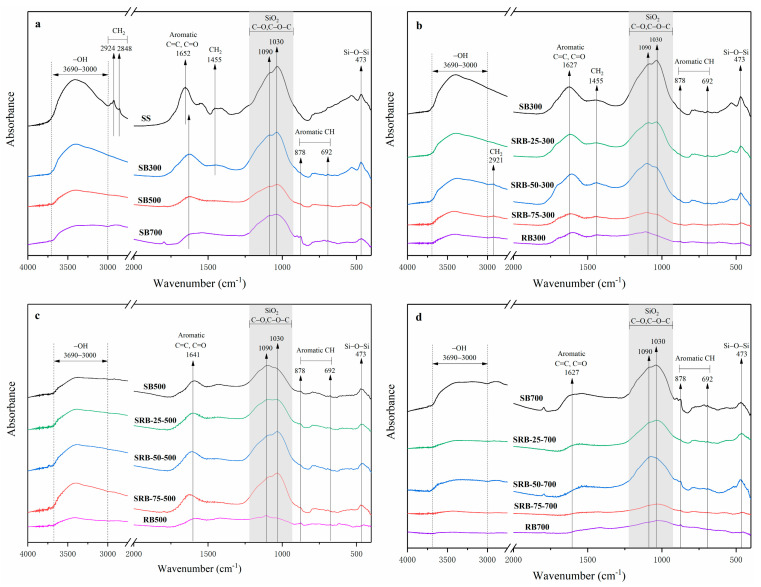
FTIR spectra and spectroscopic assignment of biochars derived from the pyrolysis of SS (**a**) and SS-RD blends at different temperatures ((**b**) 300 °C, (**c**) 500 °C, and (**d**) 700 °C).

Figure 3 shows the FTIR spectra of biochars derived from SS, RD, and SS-RD blends. The peak range of 3000–3690 cm^−^^1^ corresponded to the stretching vibration of -OH [24]. The peaks of 2848–2924 cm^−1^ and 1455 cm^−1^ were attributed to the vibration of aliphatic CH_2_, and their peaks gradually weakened with the increase of temperature (Figure 3a), indicating that the organic aliphatic hydrocarbons in SS were gradually decomposed during pyrolysis [34]. The peak of ~1652 cm^−1^ represented the C=C and C=O bond of aromatic hydrocarbons, and its peak value decreased with the increase of temperature. The further preservation of this structure also implied the formation of condensed aromatic structures in biochar [34]. The peaks at 1030 cm^−1^ and 1090 cm^−1^ were related to the stretching of C-O and C-O-C in cellulose and hemicellulose [35]. These peaks still remained in biochars derived at high temperature (Figure 3a), because the peak at ~1035 cm^−1^ also belongs to the silica absorption zone and it was gradually enriched in biochar [36]. The increasing vibration peak of Si-O-Si at 473 cm^−1^ also confirmed this conclusion. With the increased addition amount of RD, the peaks at 473 cm^−1^ and 1035 cm^−1^ gradually became weaker (Figure 3b–d) due to the dilution effect caused by the low content of SiO_2_ crystal in RD. The aromatic C-H bond was located at 692 cm^−1^ and 878 cm^−1^, and its peak intensity gradually became stronger with the increase of temperature, indicating that the aromatic structure of SBs gradually increased, which was consistent with the results in Table S1. With the increase of the RD addition, the vibration peaks of -OH, C=C, and C-O gradually weakened at the same temperature (Figure 3b–d), while the C-H peak assigned to the aromatic group gradually increased, which was consistent with the results obtained from the co-pyrolysis of the sludge and rice husk [33], indicating that the addition of RD promoted the stability of biochar. When the mixing ratio was greater than 50%, the obvious aliphatic hydrocarbon CH_2_ (2921 cm^−1^) appeared in SRB-300, which may be related to the high content of aliphatic hydrocarbon side chain in RD [37]. 

**Figure 4:**
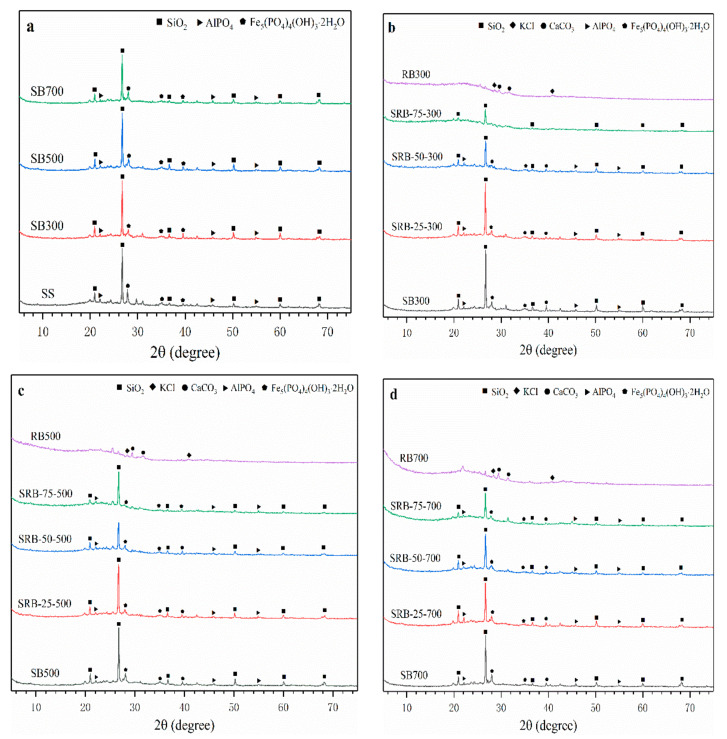
XRD patterns of biochars derived from the pyrolysis of SS (**a**) and SS-RD blends at different temperatures ((**b**) 300 °C, (**c**) 500 °C, and (**d**) 700 °C).

The mineral crystal structures in SBs, RBs, and SRBs are shown in Figure 4. The main crystal structures in SS, SBs, and SRBs were SiO_2_, iron hydroxyl-phosphates (Fe_5_(PO_4_)_4_(OH)_3_·2H_2_O), and aluminum phosphate (AlPO_4_). With the increase of temperature, the XRD patterns of SBs hardly changed, indicating that the crystal structures in SS were recalcitrant to the pyrolysis treatment at 300–700 °C. The main crystal structure of RBs was KCl and calcite (CaCO_3_), which was consistent with the crystal structure of minerals in biochars derived from wetland plants [38]. The crystal structure of SRBs was similar to that of SBs at the same pyrolysis temperature (Figure 4b–d), which was consistent with the previous results of biomass and sludge co-pyrolysis [14], indicating that the addition of RD did not cause significant changes in the crystal distribution of biochar. With the increase of RD addition from 25 wt.% to 75 wt.%, the peak strength of each crystal structure weakened due to the absence of SO_2_, Fe_5_(PO_4_)_4_(OH)_3_·2H_2_O and AlPO_4_ crystals in RD. 

### 3.3. Transformation of P during the Co-Pyrolysis Process

#### 3.3.1. Recovery of P in Biochars

As presented in Table 1, the TP content in SS (21.0 mg g^−1^) was much higher than that in RD (0.39 mg g^−1^). With the increase of pyrolysis temperature, the TP content of SBs and RBs increased from 29.8 mg g^−1^ and 0.91 mg g^−1^ to 33.0 mg g^−^^1^ and 1.32 mg g^−1^, respectively, due to the concentration effect caused by polymer decomposition and residual phosphorus in solid products [39]. The phosphorus recovery rates of SBs and RBs were 96.0–99.2% and 87.3–98.2%, respectively, which was consistent with previous studies [39,40], indicating that pyrolysis could achieve a very high phosphorus recovery rate. The unrecovered P could be ascribed to the migration of P to the liquid products during pyrolysis, which was reported in the previous study [41]. The TP contents in biochars derived from 25, 50, and 75 wt.% RD were 24.5–27.5 mg g^−1^, 17.9–21.1 mg g^−1^, and 10.1–12.4 mg g^−1^, respectively, which were lower than that in SBs because of the dilution effect of low content in *Phragmites australis*. It was noted that the phosphorus recoveries of SRBs at corresponding mixing ratios (25, 50, and 75 wt.% RD) were 92.4–96.6%, 89.1–92.3%, and 81.8–88.8%. This was lower than the recovery rate of TP in SBs at the same temperature, and the difference increased with the increase of the mixing ratio, which was consistent with a previous study [1], indicating that the co-pyrolysis of SS and RD may promote the migration of phosphorus to the liquid products.

#### 3.3.2. Transformation of P

**Figure 5:**
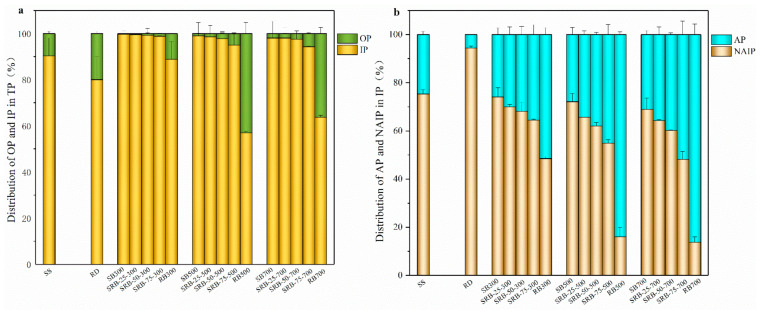
The percentage of phosphorus species in SS, RD, and biochars derived from the pyrolysis of SS, RD, and SS-RD blends by SMT method. (**a**) IP and OP; (**b**) AP and NAIP.

The distribution of phosphorus species in SS, RD, and biochar samples by the SMT method is presented in Figure 5. The proportion of IP in SS was 90.3%, indicating that a large amount of phosphate combined with metals in the sewage sludge. According to the chemical extraction (Table 1) and XRD (Figure 4) results, NAIP such as Fe_5_(PO_4_)_4_(OH)_3_·2H_2_O and AlPO_4_ was the dominant P species in sewage sludge owing to the addition of aluminum/iron-based coagulants during the wastewater treatment process, which is consistent with the previous studies [39,42]. Similarly, NAIP was identified as the main P species in *Phragmites australis* biomass. It is noteworthy that the proportion of OP in SBs (0.27–1.95%) was significantly lower than that in SS (9.68%) (*p* < 0.05), indicating that organic P in sewage sludge was converted into inorganic P during pyrolysis. This phenomenon could be attributed to the low thermostability of organic P such as phytate, which could be converted into orthophosphate via degradation of the linked organic groups or pyrophosphate by dehydration during pyrolysis [43,44,45]. With increasing pyrolysis temperature from 300 to 700 °C, the difference between IP proportions in SBs (98.0–99.7%) was not significant (*p* > 0.05), implying that the P transformation mainly occurred between different forms of inorganic P. Huang and Tang also reported that long-chain polyphosphates and orthophosphate in sewage sludge were converted into short-chain polyphosphates and pyrophosphate after pyrolysis at 250–600 °C [44]. While for RBs, the proportion of IP decreased from 88.9% to 57.0% and 63.7% with increasing temperature from 300 to 500 and 700 °C, in accordance with the transformation route in the pyrolysis of wheat straw and peanut husks [40]. With the increasing in the RD mixing ratio from 25 wt.% to 75 wt.%, the IP content in SRBs significantly declined from 21.1–25.9 mg g^−1^ to 9.69–11.7 mg g^−1^ regardless of the co-pyrolysis temperature (*p* < 0.05) owing to the low IP content in *Phragmites australis* biomass (Table 1). However, the difference between IP proportions in SRBs with different RD mixing ratios was not significant (*p* > 0.05) (Figure 5a), highlighting that the addition of RD had no obvious effect on the distribution of IP and OP in SRBs. 

Considering the predominant role of IP in SS, RD, and the derived biochars, the variation tendencies of AP and NAIP were further investigated. With elevation in the pyrolysis temperature, the proportion of NAIP in SBs (68.9–74.1%) slightly changed, which was similar to that in SS (75.4%). This implies that NAIP such as Fe_5_(PO_4_)_4_(OH)_3_·2H_2_O and AlPO_4_ was still the dominant P species in the sewage sludge derived biochars regardless of pyrolysis temperature, which was confirmed in the XRD patterns (Figure 4). However, the proportion of AP in RB500 (83.9%) and RB700 (86.2%) was significantly higher than that in RD (5.6%) and RB300 (51.5%) (*p* < 0.05), indicating that a higher pyrolysis temperature enhanced the transformation of NAIP to AP in *Phragmites australis* derived biochar, making stable Ca-P species become the main P species. This transformation mainly resulted from the abundant Ca content in *Phragmites australis* and the better stability of the Ca-P species, and thus the original NAIP in biomass decomposed and formed stable Ca-P compounds at high pyrolysis temperature. With increasing the RD mixing ratio from 25 wt.% to 75 wt.%, AP and NAIP contents in SRBs significantly declined regardless of the co-pyrolysis temperature (*p* < 0.05) due to the low AP and NAIP contents in *Phragmites australis* (Table 1). It was noteworthy that the proportion of AP in SRBs was higher than that in SBs derived at the same pyrolysis temperature, and the difference was enlarged at a higher RD mixing ratio (Figure 5b), suggesting that the co-pyrolysis of SS and RD promoted the transformation of NAIP to AP. It was reported that the addition of straw and CaO promoted the formation of Ca_3_(PO_4_)_2_ in sludge-derived biochar [46,47]. Therefore, *Phragmites australis* can serve as a Ca source for promoting the generation of Ca-P compounds in biochar during co-pyrolysis with sewage sludge.

OP and NAIP are considered as potentially bio-available P [48,49]. The OP+NAIP content in SBs (13.6–15.7 mg g^−1^) was much higher than that in SS (10.2 mg g^−1^) (Table 1), indicating that pyrolysis could improve the concentration of available phosphorus in biochar, in agreement with the results reported by Qian and Jiang [39]. With the increase of RD addition from 25 wt.% to 75 wt.%, the total content of OP and NAIP in SRBs significantly decreased from 10.5–12.7 mg g^−1^ to 4.59–5.37 mg g^−1^ (*p* < 0.05), indicating that excessive RD could reduce the available phosphorus content in biochar. Considering that the total content of OP and NAIP in biochars derived from the 25 wt.% RD group was higher than that in SS, 25 wt.% RD could be an adequate mixing ratio of co-pyrolysis for the production of biochar with high bio-available P content. Moreover, in comparison with SBs, the SRBs contained a higher proportion of AP, implying that co-pyrolysis with *Phragmites australis* favored the formation of long-term plant-available P and alleviated the risk of P leaching loss.

### 3.4. Removal of Heavy Metals

#### 3.4.1. Total Contents of Heavy Metals in Biochars

Table 2 lists the total concentrations of Cr, Ni, Cu, Zn, and Pb in SS, RD, and biochar samples. The threshold values of heavy metals cited from the control standards of pollutants for agricultural sludge in China (GB 4284-2018) are also listed in Table 2. The content of Zn in sludge was the highest, followed by Cu, Cr, Ni, and Pb. With the increase in temperature, the content of heavy metals in SBs gradually increased, indicating that heavy metals were gradually accumulated in biochar during pyrolysis [50]. The total content of heavy metals (except Cr) in SRBs decreased with the increase in the mixing ratio (Table 2). This can be explained by the dilution effect, since RD contained much lower heavy metals than SS. The content of Cr in SRBs (215–377 mg kg^−1^) was higher than that in SBs (261–290 mg kg^−1^), because the Cr in RD (93.9 mg kg^−1^) was close to that in sludge (182 mg kg^−1^), but the heavy metals concentration rate was high due to the large weight loss during RD pyrolysis (Figure 1). The recovery rate of heavy metals in SBs was 93.3–103% (Table S2), which was consistent with previous studies [32], indicating that heavy metals were fixed in biochar during pyrolysis. The recovery rate of Pb in SRBs (90.4–93.3%/89.5–94.0%/72.0–92.0%) was lower than that in SBs (93.3–94.1%) at the same temperature, indicating that adding RD could promote the volatilization of Pb during SS pyrolysis [20]. The contents of Cr, Ni, Cu, Zn, and Pb in all biochar were lower than the heavy metals threshold of grade B sludge products, indicating that biochar produced by pyrolysis could be used in any arable land except for food crops. It was noteworthy that the total concentration of Zn in SBs (1218–1372 mg kg^−1^) was higher than the heavy metals threshold of grade A sludge products (1200 mg kg^−1^). However, the total Zn content of SRBs was lower than the heavy metals threshold of grade A sludge products, suggesting that the co-pyrolysis of RD and SS would improve the land-use grade of biochar.

#### 3.4.2. Transformation of Zn

**Figure 6:**
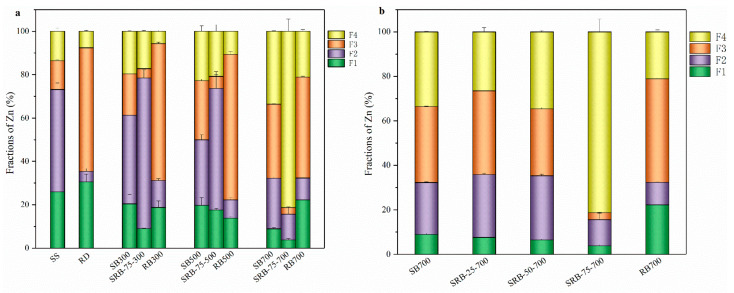
Fraction distribution of Zn in biochars. (**a**) raw material and biochars derived from the pyrolysis of SS, RD, and 75 wt.% RD; (**b**) biochars derived from SS-RD blends at 700 °C.

The speciation of heavy metals was one of the most critical factors affecting their bioavailability [20]. According to the above section, the content of Zn in sludge was the highest, and the concentration of Zn in SBs exceeds the threshold of Zn for grade A sludge products. The chemical forms of Zn in SS, RD, and biochar samples are measured according to the sequential extraction procedure (Table S3), and their fraction distribution is shown in Figure 6. Among the chemical forms of heavy metals, the exchangeable (F1) and reducible (F2) states were the most unstable, which were easily absorbed by plants or water systems and lead to pollution, and were considered as direct toxicity. The oxidizable state (F3) was relatively stable, leading to the release of soluble metals only under oxidizing conditions and is considered potentially toxic. The residue state (F4) was very stable and was generally not considered toxic. The F1+F2 proportion of Zn in SS was as high as 73.2%, which indicates that there was a high environmental risk when directly applying sludge to soil [51]. With the increase of temperature, the F1 + F2 proportion of Zn in SBs and RBs decreased, while the proportion of F4 increased, indicating that the pyrolysis process would reduce the toxicity of heavy metals [52]. It is noteworthy that the proportion of F4 in SRB-75-700 (81.3%) was much higher than that in SB700 (33.6%) and RB700 (21.1%), which was consistent with previous studies, indicating that adding RD at high temperature was more conducive to the stability of heavy metals [16,53]. With the increase of RD addition, the proportion of F1, F2, and F3 of Zn in SRBs decreased, while the proportion of F4 increased (Figure 6b), indicating that the stability of heavy metals increased with the increase of RD addition during the co-pyrolysis of SS.

## 4. Conclusions

The co-pyrolysis of sewage sludge and *Phragmites australis* had an obvious effect on the characteristics of biochar and the behaviors of P and heavy metals. Compared with SBs, SRBs had lower yield and ash content, and higher pH, C content and aromatic structure, indicating that co-pyrolysis was beneficial to improve the carbon fixation capacity and stability of biochar. With the increase of the pyrolysis temperature, the contents of TP and heavy metals in SBs, SRBs, and RBs increased. The phosphorus content of SRBs was lower than that of SBs at the same temperature, but the addition of RD promoted the transformation of NAIP to AP. In addition, a higher RD mixing ratio greatly reduced the heavy metals content and environmental risk of biochar, and improved its land use potential. Considering the increasing sewage sludge production worldwide and stringent disposal regulations, co-pyrolysis is a promising scheme to achieve safer treatment of sewage sludge and to produce value-added biochar. The derived P-enriched biochar can be utilized as a potential ameliorant to improve soil fertility, alleviating the crisis of phosphate rock shortage worldwide. Furthermore, the soil application of sewage sludge derived biochar by co-pyrolysis should be further investigated.

## Figures and Tables

**Table 1:** Amount of P (TP, IP, OP, NAIP, and AP) in SS, RD, and biochars derived from the pyrolysis of SS, RD, and SS-RD blends.

Sample	TP	IP	OP	NAIP	AP	TP Recovery (%)
	(mg g^−1^)					
SS	21.0 ± 0.2	15.8 ± 1.4	1.69 ± 0.17	8.53 ± 0.20	2.79 ± 0.16	
RD	0.39 ± 0.04	0.16 ± 0.02	0.04 ± 0.00	0.17 ± 0.00	0.01 ± 0.00	
SB300	29.8 ± 0.6	25.7 ± 0.4	0.07 ± 0.05	13.5 ± 0.7	4.72 ± 0.52	99.2
SB500	30.9 ± 0.1	26.1 ± 1.5	0.27 ± 0.04	14.7 ± 0.7	5.69 ± 0.59	96.0
SB700	33.0 ± 1.2	27.7 ± 2.1	0.55 ± 0.05	15.2 ± 1.1	6.85 ± 0.35	96.0
SRB-25-300	24.5 ± 0.8	21.1 ± 0.1	0.09 ± 0.00	10.4 ± 0.2	4.47 ± 0.47	96.6
SRB-25-500	26.9 ± 1.1	23.7 ± 1.2	0.35 ± 0.00	11.1 ± 0.0	5.82 ± 0.25	96.4
SRB-25-700	27.5 ± 1.7	25.9 ± 1.2	0.51 ± 0.03	12.2 ± 0.1	6.79 ± 0.61	92.4
SRB-50-300	17.9 ± 0.2	15.3 ± 0.5	0.11 ± 0.04	7.89 ± 0.41	3.69 ± 0.39	92.3
SRB-50-500	19.9 ± 1.3	19.4 ± 0.6	0.45 ± 0.06	8.14 ± 0.19	4.98 ± 0.11	90.4
SRB-50-700	21.1 ± 1.7	19.7 ± 0.7	0.49 ± 0.00	9.18 ± 0.02	6.08 ± 0.11	89.1
SRB-75-300	10.1 ± 1.0	9.69 ± 0.02	0.12 ± 0.01	4.47 ± 0.03	2.46 ± 0.28	88.8
SRB-75-500	11.0 ± 0.2	10.1 ± 0.5	0.53 ± 0.03	4.53 ± 0.12	3.72 ± 0.34	81.8
SRB-75-700	12.4 ± 0.2	11.7 ± 0.0	0.71 ± 0.03	4.66 ± 0.32	5.01 ± 0.55	83.3
RB300	0.91 ± 0.07	0.80 ± 0.07	0.10 ± 0.00	0.32 ± 0.01	0.34 ± 0.02	87.3
RB500	1.21 ± 0.04	0.61 ± 0.01	0.46 ± 0.05	0.09 ± 0.02	0.47 ± 0.01	98.2
RB700	1.32 ± 0.03	0.79 ± 0.01	0.45 ± 0.03	0.08 ± 0.01	0.50 ± 0.03	96.8

**Table 2:** Total concentrations of heavy metals in samples and their threshold values in the control standards of China.

Sample	Cr	Ni	Cu	Zn	Pb
	(mg kg^−1^)				
SS	182 ± 2	64.3 ± 1.4	122 ± 1	829 ± 12	53.4 ± 2.0
RD	93.9 ± 8.6	7.78 ± 2.7	9.07 ± 3.19	37.7 ± 5.0	1.04 ± 0.12
SB300	261 ± 1	88.9 ± 1.2	169 ± 1	1218 ± 18	71.7 ± 0.1
SB500	272 ± 2	95.6 ± 1.6	182 ± 5	1289 ± 9	77.2 ± 1.1
SB700	290 ± 6	100 ± 2	192 ± 3	1372 ± 29	81.5 ± 0.9
SRB-25-300	284 ± 4	85.1 ± 0.0	148 ± 9	987 ± 5	60.1 ± 0.6
SRB-25-500	360 ± 10	104 ± 3	165 ± 3	1110 ± 6	66.1 ± 0.4
SRB-25-700	371 ± 3	104 ± 2	175 ± 0	1188 ± 5	68.4 ± 0.4
SRB-50-300	263 ± 7	72.2 ± 1.9	113 ± 2	791 ± 2	46.4 ± 0.2
SRB-50-500	307 ± 5	78.3 ± 1.7	125 ± 3	879 ± 20	52.1 ± 1.0
SRB-50-700	377 ± 8	87.6 ± 0.7	141 ± 2	984 ± 6	53.9 ± 0.6
SRB-75-300	215 ± 6	44.0 ± 4.6	75.1 ± 2.8	511 ± 3	26.8 ± 0.2
SRB-75-500	277 ± 3	45.6 ± 2.4	85.7 ± 2.5	589 ± 25	31.4 ± 1.4
SRB-75-700	324 ± 3	53.5 ± 4.1	93.3 ± 5.9	645 ± 3	27.4 ± 0.0
RB300	222 ± 4.	15.1 ± 7.1	17.9 ± 1.4	77.1 ± 1.2	2.51 ± 0.09
RB500	306 ± 7	33.8 ± 2.1	31.0 ± 3.7	103 ± 3	3.65 ± 0.52
RB700	378 ± 4	26.3 ± 1.6	33.0 ± 2.9	105 ± 2	2.66 ± 0.08
Threshold values ^a^					
Grade A	500	100	500	1200	300
Grade B	1000	200	1500	3000	1000

^a^ According to the Agricultural Sludge Pollutant Control Standard (GB 4284-2018): Grade A can be applied to the arable, garden, and pasture fields, while Grade B cannot be used to grow food crops.

## Data Availability

The datasets generated for this study are available on request to the corresponding author.

## Supplementary Materials

The following supporting information can be downloaded at: https://www.mdpi.com/1660-4601/19/5/2818/s1, Table S1: Basic physicochemical properties of SS, RD, and biochars derived from SS, RD, and SS-RD blends; Table S2: Recovery of heavy metals in biochars derived from SS, RD, and SS-RD blends; Table S3: Chemical forms of Zn in SS, RD, and biochars.
