# Supplementary material for: Co-Pyrolysis of Sewage Sludge and Wetland Biomass Waste for Biochar Production: Behaviors of Phosphorus and Heavy Metals
Source: Int J Environ Res Public Health. 2022 Feb 28;19(5):2818. doi: 10.3390/ijerph19052818 (PMC8909961; doi:10.3390/ijerph19052818)
Supplement: Supplementary file 1 [file ijerph-19-02818-s001.zip › ijerph-1558901-supplementary.pdf]

Co-pyrolysis of sewage sludge and wetland biomass waste for biochar production: behaviors of phosphorus and heavy metals

Ilham Gbouri [1, †], Fan Yu [1, 2, †], Xutong Wang [1], Junxia Wang [1], Xiaoqiang Cui [1, *], Yanjun Hu [2], Beibei Yan [1], Guanyi Chen [1, 3]

[1] *School of Environmental Science and Engineering/ Tianjin Key lab of Biomass Waste Utilization, Tianjin University, Tianjin 300072, China*

[2] *Institute of Energy and Power Engineering, Zhejiang University of Technology, Hangzhou 310023, China*

[3] *School of Mechanical Engineering, Tianjin University of Commerce, Tianjin, 300134, PR China*

Corresponding Authors:

* Xiaoqiang Cui, Tel: +86-022-87401929, E-mail: cuixiaoqiang@tju.edu.cn

[†] The first two authors contribute to the work equally.

Table S1. Basic physicochemical properties of SS, RD, and biochars derived from SS, RD and SS-RD blends.

Samples	pH	Ash (%)	Elemental content (%)				Atomic ratio		
			C	H	N	O	H/C	O/C	(O+N)/C
SS	6.90	49.6	21.2	3.80	3.33	22.1	2.16	0.78	0.92
RD	5.10	5.40	45.1	5.31	0.80	43.4	1.41	0.72	0.74
SB300	7.06	72.6	17.7	3.39	2.82	3.50	2.30	0.15	0.28
SB500	8.56	76.8	15.2	0.96	6.07	0.90	0.76	0.04	0.39
SB700	9.69	81.4	14.2	0.44	2.27	1.70	0.37	0.09	0.23
SRB-25-300	7.22	62.5	22.3	1.49	2.77	10.9	0.80	0.37	0.47
SRB-25-500	8.88	68.3	22.9	1.15	2.21	5.50	0.60	0.18	0.26
SRB-25-700	10.2	73.6	21.6	0.53	1.48	2.90	0.29	0.10	0.16
SRB-50-300	8.44	51.3	32.1	2.10	2.36	12.1	0.78	0.28	0.35
SRB-50-500	9.17	60.1	31.9	1.36	1.97	4.60	0.51	0.11	0.16
SRB-50-700	11.2	63.1	31.7	0.62	1.36	3.20	0.24	0.08	0.11
SRB-75-300	8.96	34.9	47.7	2.88	1.96	12.5	0.72	0.20	0.23
SRB-75-500	10.3	38.5	49.2	1.87	1.81	8.70	0.46	0.13	0.16
SRB-75-700	11.7	46.2	44.0	0.74	1.26	7.80	0.20	0.13	0.16
RB300	7.16	15.3	63.2	3.40	1.48	16.6	0.65	0.20	0.22
RB500	8.43	18.0	69.4	2.27	1.40	8.90	0.39	0.10	0.11
RB700	9.14	15.3	73.5	1.07	1.17	8.90	0.17	0.09	0.10

Table S2. Recovery of heavy metals in biochars derived from SS, RD, and SS-RD blends.

Sample	Cr	Ni	Cu	Zn	Pb
	Recovery（%）				
SB300	100	96.8	96.9	103	94.1
SB500	97.5	96.9	97.5	101	94.3
SB700	97.3	95.5	96.1	101	93.3
SRB-25-300	111	106	98.3	97.7	93.1
SRB-25-500	128	118	100	100	93.3
SRB-25-700	124	110	99.5	100	90.4
SRB-50-300	105	111	94.8	101	94.0
SRB-50-500	108	105	92.7	98.5	93.0
SRB-50-700	124	110	97.4	103	89.5
SRB-75-300	89.9	97.6	97.8	105	92.0
SRB-75-500	98.0	85.5	94.5	103	91.4
SRB-75-700	104	90.8	93.0	102	72.0
RB300	88.4	72.4	73.6	76.4	90.4
RB500	103	137	108	86.1	111
RB700	115	96.8	104	79.3	73.4

Table S3. Chemical forms of Zn in SS, RD, and biochars.

Sample	Zn ($mg \cdot kg^{-1}$)					Recover (%)
	F1	F2	F3	F4	Total	
SS	193 ± 6	349 ± 22	98.0 ± 2.6	101 ± 11	740	89.3
RD	10.1 ± 1.2	1.57 ± 0.44	18.8 ± 0.0	2.50 ± 0.14	32.9	87.3
SB300	208 ± 42.9	416 ± 44	194 ± 10	200 ± 2	1019	83.6
SB500	206 ± 36	315 ± 24.1	284 ± 8	238 ± 26	1043	80.9
SB700	105 ± 5	272 ± 3	399 ± 2	392 ± 2	1169	85.2
SRB-25-700	76.3 ± 1.6	281 ± 5	375 ± 1	264 ± 18	997	83.9
SRB-50-700	54.4 ± 1.4	236 ± 6	248 ± 4	284 ± 4	823	83.6
SRB-75-300	24.2 ± 0.4	185 ± 10	11.4 ± 0.1	45.8 ± 1.1	266	52.1
SRB-75-500	51.1 ± 1.5	163 ± 22.4	16.1 ± 2.1	60.3 ± 8.7	290	49.2
SRB-75-700	17.6 ± 2.1	53.0 ± 12.9	13.8 ± 0.9	366 ± 26	450	69.7
RB300	13.4 ± 2.1	8.91 ± 0.53	45.1 ± 0.5	4.05 ± 0.21	71.5	92.8
RB500	12.8 ± 0.6	7.83 ± 0.98	62.1 ± 1.1	9.85 ± 0.07	92.6	90.3
RB700	21.2 ± 0.0	9.57 ± 0.11	44.3 ± 0.4	20.1 ± 0.8	95.2	91.0
